# Analyzing the interaction of main components of hidden curriculum in medical education using interpretive structural modeling method

**DOI:** 10.1186/s12909-020-02094-5

**Published:** 2020-06-01

**Authors:** Yaser Sarikhani, Payam Shojaei, Mohammad Rafiee, Sajad Delavari

**Affiliations:** 1 Student Research Committee, Shiraz University of Medical Sciences, Shiraz, Iran; 2 Department of Management, School of Economics, Management and Social Sciences, Shiraz University, Shiraz, Iran; 3 Health Human Resources Research Center, School of Management and Information Sciences, Shiraz University of Medical Sciences, Shiraz, Iran

**Keywords:** Hidden curriculum, Medical education, Curriculum, Interpretive structural modeling

## Abstract

**Background:**

Hidden curriculum (HC) is considered as unintended learning experiences in medical education (ME). This may include values, norms, beliefs, skills, and knowledge which could potentially influence learning outcomes. HC has key components that must be identified and considered properly by individuals and organizations involved in ME.

**Objectives:**

This study aimed to determine the main components of hidden curriculum in medical education (HCME) and the interrelationships among them.

**Methods:**

In this mixed-method study initially we performed a scoping review and determined the main components of HCME using qualitative content analysis approach. Then, the interrelationships among these components were investigated using Interpretive Structural Modeling (ISM).

**Results:**

Ten key components for HCME were identified in scoping review. We classified them into four main categories including structural, educational, cultural, and social factors. The ISM analysis revealed that organizational rules and structure, dominant culture of educational environments, teaching and assessment approaches, as well as clinical and educational physical setting were the independent or driving factors. While, social components were dependent and influenced by basic components.

**Conclusion:**

The ISM model indicated that role modeling behaviors and interpersonal relationships (social factors) are under influence of underlying organizational and educational factors. These results should be considered at all stages of educational management including planning process, implementation of the programs, and development of formal curricula. According to the importance of contextual factors, components of HC must be analyzed and interpreted based on the specific conditions of each educational institution.

## Background

Curriculum is one of the key components of medical education (ME) [1]. Success in achieving educational goals largely depends on the quality of the curriculum [2]. Learning experiences of medical students’ are not limited to formal curriculum; rather, there are many other factors that are shaping students’ experiences and skills [3]. These factors that are known as ‘Hidden Curriculum’ (HC) includes a set of values, behavioral norms, attitudes, skills, and knowledge that medical students learn implicitly [4].

HC has several effects on various groups such as students, physicians and teachers, patients, as well as administrative personnel [5]. However, the impact of HC on medical students is of great importance to health systems [6]. HC could determine various aspects of students’ future clinical practice [7] such as medical professionalism [8, 9], and medical competency [10]. This also has a significant impact on their ethical performance in relation to the patients [11, 12].

Due to the desirable and negative effects of HC on healthcare processes and outcomes, it seems necessary to identify its components [8, 13]. Gaufberg et al. have noted that organizational, cultural, and structural factors are among the most important components of HC [14]. Another study conducted in Iran showed that educational, social, physical, and organizational factors are the main components of HC in the (HCME) [15].

Identification of the main components of HC could be of great help in maximizing its desirable values, while decreasing its unfavorable results [16]. Interpretive structural modeling (ISM) is a technique that can convert an obscure and poorly expressed mental model into a transparent and well-defined format useful for many purposes such as planning, decision making, and applying necessary changes in the processes [17]. Based on our searches, no previous studies have used this technique for analysis of components of HCME. Therefore, the first objective of this study was to determine the leading components of HCME. Secondly, this study aimed to determine the structural interrelationships among these components using ISM in order to provide a deeper insight into the concept.

## Methods

We conducted this mixed-method study in two main phases. First, we performed a scoping review to identify the main components of HCME. Scoping review -in contrary with systematic review- allows us to include studies with heterogeneous samples and designs; while, quality assessment of each article is not assumed as an inclusion criterion. Thus, this type of review provides us with opportunity to identify the main components and characteristics of a concept [18]. We searched five online databases including PubMed, Scopus, Sciencedirect, Web of Science, and Scientific Information Database (SID) for related studies. We used “curriculum”, “curricula”, “hidden”, “informal”, “implicit”, “tacit”, “medical”, and “medical education” as search terms. We combined search results using “AND” and “OR” logical operators in order to achieve the outcome of interest. We only included English language articles in the final analyses because of translation limitations. Articles were selected according to the three main criteria of scoping review studies. In this regard, we considered the main components, hidden curriculum, as well as medical education as “Population, Concept, and Context” (PCC) respectively. Table 1 shows the search strategy of the study.

**Table 1:** Search strategy for the main components of hidden curriculum in medical education

Searched Databases	PubMed, Scopus, Sciencedirect, Web of Science, and Scientific Information Database (SID)
**Search strategy**	**#1 AND #2 AND #3**
**#1**	Curriculum OR Curricula
**#2**	Hidden OR Informal OR Implicit OR Tacit
**#3**	Medical OR “Medical Education”
**Limitations**	Language: articles with at least an abstract in English

Using the search strategy mentioned above, a total of 583 articles were extracted from all databases. After removing duplicates, 407 papers were selected for the assessment phase. In order to select relevant articles, we performed an iterative three-step appraisal process; so that in each step we modified the search strategy, searched the databases, and reviewed new papers. The objective of the appraisal phase was to identify articles that determined and explained the components of HCME. Two reviewers screened the extracted articles independently at three levels. At the first step, they screened titles of the articles independently. At this step the researchers selected 94 articles for further appraisal. Then, two reviewers scanned the abstracts and excluded those were not consistent with the objective appraisal process. At this step, 40 full-text articles were selected for further evaluation. Eventually, the reviewers apprised full-texts and 14 studies were included in the final analysis. At all stages of screening, a third researcher reviewed cases of disagreement.

We applied a qualitative content analysis to synthesis data extracted from articles [18]. In this regard, we used a qualitative thematic analysis to develop categories and their related sub-categories [19]. At the first step, we specified preliminary codes to develop main themes regarding the main components of HCME. Then we carried out an interpretive analysis of the initial codes and thereby determined the main categories and their related sub-categories. We classified data extracted from included studies into 10 key components and four main categories. The components identified in this step were used in the quantitative analysis. In order to increase comprehensibility of the concepts and to provide a consensus on the emerged themes, all participants attended a discussion panel prior to the quantitative phase and approved the components and the main categories.

In the quantitative phase of the study we used the ISM technique to determine direct relationships among the main components of HC and to provide a more comprehensive understanding of the concept. ISM is a technique for determining the interrelationships among specific variables that describe a subject. This methodology can explain the order and direction of complex relationships among the elements of a system. Moreover, it can develop the structural and mutual relationships among components of a system and can determine levels of these factors based on their driver or dependence power. Comprehensibility for a wide range of users, integrity in the combination of expert opinions, and applicability in the study of complex systems comprising multiple components are among the main advantages of the ISM methodology [20].

There are seven steps involved in the ISM method: 1- Determining the main variables of a subject. 2- Developing Structural Self-Interaction Matrix (SSIM) for variables. This matrix indicates pair-wise relationships among variables and components. 3- Developing Reachability Matrix (RM) from the SSIM and checking the matrix for transitivity (The transitivity of the relation states that if variable A is related to B and B is related to C, then A is related to C). 4- Partitioning the RM into different levels. 5- Drawing a directed graph based on the relationships and removing the transitive links. 6- Converting the resultant diagram into the ISM model. 7- MICMAC analysis in order to determine driving and dependence components [21].

Given that the ISM technique is based on the experts’ opinions, all nine faculty members of the Medical Education Department at Shiraz University of Medical Sciences participated in the second stage of the study. All participants were completely familiar with the topic and completed an informed consent form before entering the study.

In order to obtain experts’ opinions, each participant completed a SSIM. SSIM allows for pair-wise comparison of the components. Based on the existence and direction of the relationships between any two factors (*row: i* and column: *j*), we used four following symbols for mutual comparison: “*X”* when *i* and *j* will influence each other, “*O”* when *i* and *j* are unrelated, *“A”* when *i* will be influenced by *j*, and “*V”* when *i* will influence *j*. Finally, we developed the integrated SSIM using the mode of experts’ opinions (Table 3).

**Fig. 1:**
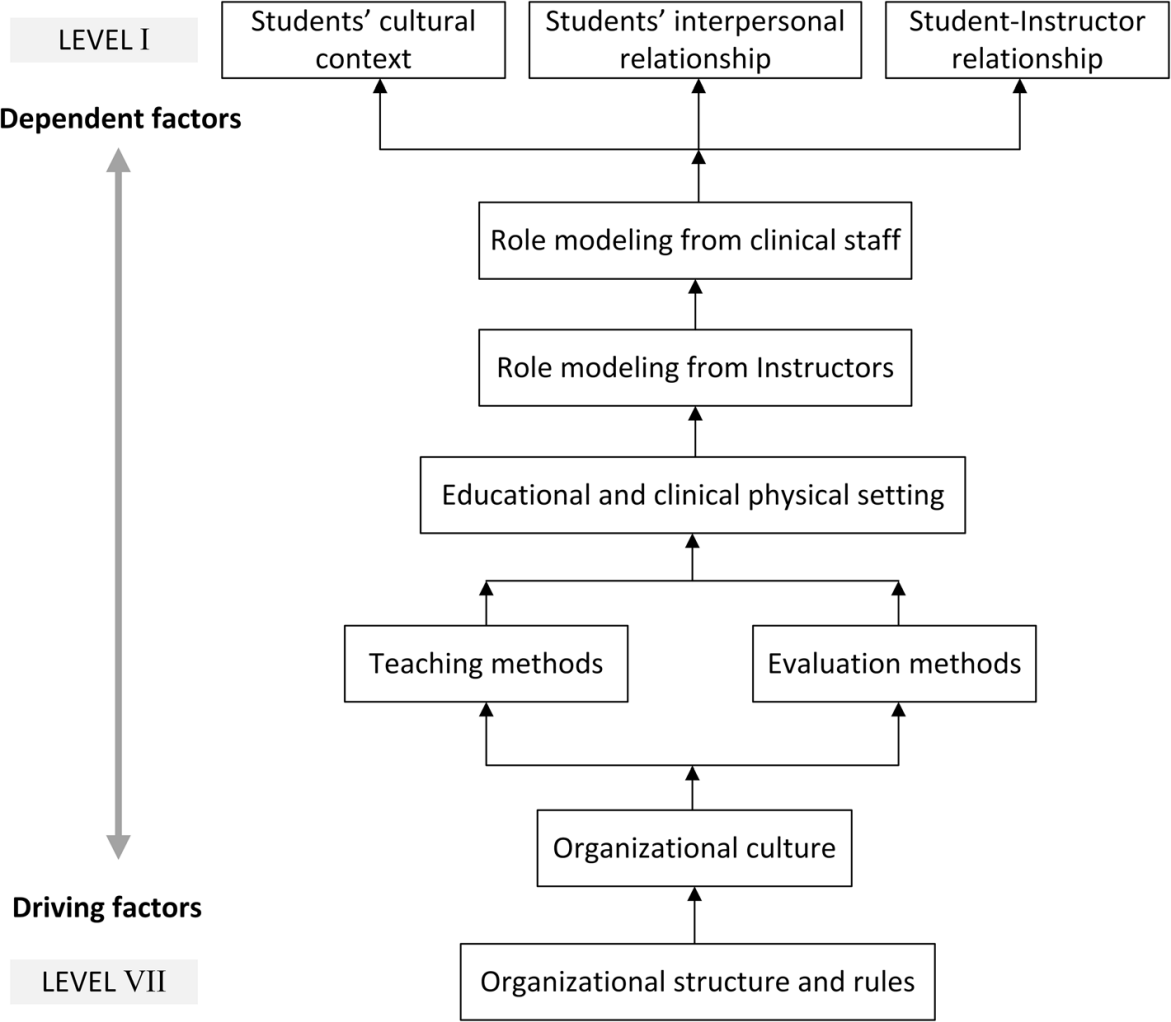
ISM model of components of HC in medical education

At the second step of the ISM technique, we converted the SSIM into the initial reachability matrix (IRM). For this purpose, we substituted symbols of SSIM by 0 s or 1 s in the IRM as follows: When the SSIM (*i, j*) entry was “*X”* both (*i, j*) and (*j, i*) entry in the IRM became 1. When the SSIM (*i, j*) entry was “*O”* both (*i, j*) and (*j, i*) entry in the IRM became 0. When the SSIM (*i, j*) entry was “*A”* the (*i, j*) and (*j, i*) entry in the IRM became 0 and 1 respectively. When the SSIM (*i, j*) entry was “*V”* the (*i, j*) and (*j, i*) entry in the IRM became 1 and 0 respectively (Table 4). Thereafter, we developed the IRM and then the final reachability matrix (FRM) emerged by including 1* entries to incorporate transitivity (Table 5). By incorporating transitivity, we filled the gap, if any, in the opinions gathered during development of the SSIM. We extrapolated the FRM through checking the IRM for the transitivity rule and we updated the matrix till full transitivity was established. At this step, we calculated driving power (number of 1’s in each factor’s row) and dependence power (number of 1’s in each factor’s column) for each factor. We used these data for developing the ISM diagram and the MICMAC (Cross Impact Matrix-Multiplication Applied to Classification) matrix. The preliminary ISM diagram emerged from the final reachability matrix containing transitive links. Then, the final diagram was generated after removing indirect relations. This diagram shows interdependency of the factors (Fig. 1).

Level partitioning was the next step of the ISM technique which was essential for developing the ISM diagram and model. At this step, we determined the antecedent set and the reachability set of each factor from the FRM. The antecedent set consisted of a factor and the other factor that may affect it, while the reachability set consisted of a factor and the other factor that it may affect them. Accordingly, the intersection of these sets was derived for all factors and we specified the level of these factors. We replaced factors whose reachability and intersection sets were the same on the top level of the ISM hierarchy. These factors would not lead to other factors above their own level in the ISM hierarchy. After determining the top-level factors, they were removed from the level partitioning process. This process was repeated until the level of all factors was identified (Table 6).

**Fig. 2:**
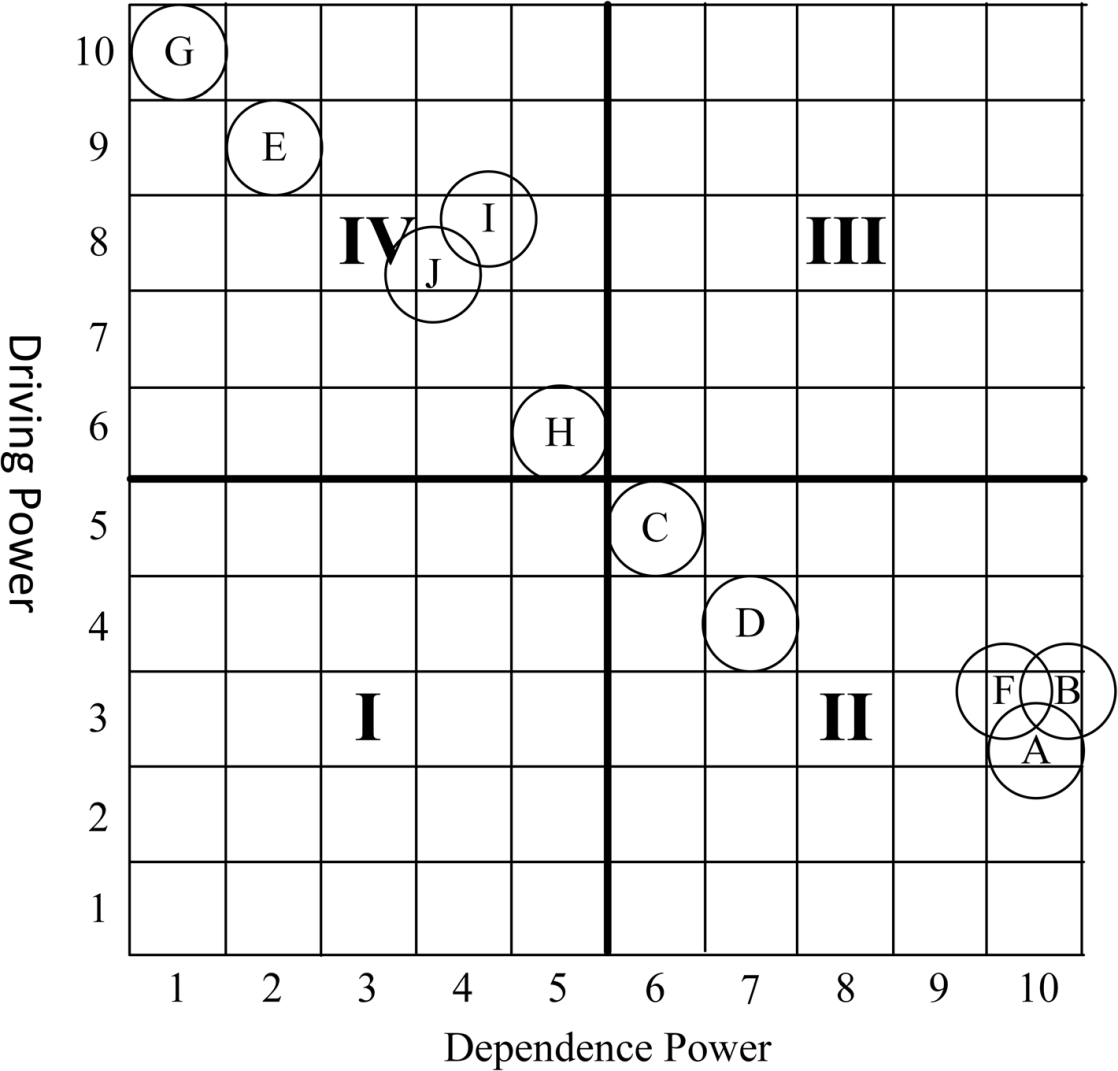
MICMAC matrix of components of HC in medical education

Finally, we analyzed the components of HC using the MICMAC matrix which is based on the dependency and driving scores of the components derived from the FRM data (Fig. 2). Using the MICMAC matrix, we classified the components of HC into four categories including independent, dependent, autonomous, and linkage factors. Independent or driving factors have strong drive power but weak dependence power (Zone IV). Dependent factors have strong dependence power but weak drive power (Zone II). Autonomous factors have weak drive power and dependence power (Zone I). Linkage factors have strong driver power and dependence power (Zone III).

## Results

At the first phase of the study we conducted a literature review in order to identify the key components of HCME. A total number of 14 studies [22–34] were included in the study. We extracted 10 main components using thematic content analysis approach and classified them into four categories including structural factors, educational factors, cultural factors, and social factors. Table 2 presents the results of thematic analysis.

**Table 2:** Main components of hidden curriculum in medical education

Category	Main Components of HC	Frequency (References)
Social Factors	(A) Student-Instructor relationships	5 (22, 24, 28, 29, 31)
	(B) Students’ interpersonal relationships	8 (13, 22, 25, 26, 28, 29, 32, 33)
	(C) Role modeling from instructors	10 (13, 22, 23, 25, 27–29, 31, 32, 34)
	(D) Role modeling from clinical staff	4 (23, 25, 29, 34)
Cultural Factors	(E) Organizational culture	7 (22, 23, 26–28, 31, 34)
	(F) Students’ cultural context	2 (23, 28)
Structural Factors	(G) Organizational rules and structures	8 (22, 23, 25, 27–29, 31, 34)
	(H) Educational and clinical physical setting	4 (13, 25, 30, 31)
Educational Factors	(I) Teaching methods	2 (26, 27)
	(J) Evaluation methods	1 (26, 33)

At the second phase, we applied ISM method with the participation of 9 ME experts. For pair-wise comparison of the components, each expert filled an SSIM and we developed the integrated SSIM accordingly (Table 3). The IRM and the FRM are presented in Tables 4 and 5, respectively. Table 6 presents the level partitioning of the components of HCME. Based on the results of the ISM model presented in the Fig. 1, the main components of HCME were classified into 7 levels. These results show that organizational rules and structures as well as organizational culture had the highest driving power among the main components of HCME respectively. Moreover, the components with highest dependence power were student-instructor relationships, students’ interpersonal relationships, and students’ cultural context.

**Table 3:** Integrated structural self-interaction matrix for components of HC

Components	J	I	H	G	F	E	D	C	B	A
**A**	*A*	*A*	*A*	*A*	*X*	*A*	*O*	*O*	*V*	
**B**	*O*	*O*	*A*	*A*	*X*	*A*	*O*	*O*		
**C**	*O*	*O*	*A*	*A*	*V*	*A*	*V*			
**D**	*O*	*O*	*A*	*A*	*V*	*A*				
**E**	*V*	*V*	*V*	*A*	*V*					
**F**	*A*	*A*	*A*	*A*						
**G**	*V*	*V*	*V*							
**H**	*A*	*A*								
**I**	*X*									
**J**										

“*X”:* when *i (row)* and *j (column)* will influence each other;

“*O”:* when *i* and *j* are unrelated;

*“A”:* when *i* will be influenced by *j*;

“*V”:* when *i* will influence *j*

**Table 4:** Initial reachability matrix for components of HC

Components	A	B	C	D	E	F	G	H	I	J
**A**	*1*	*1*	*0*	*0*	*0*	*1*	*0*	*0*	*0*	*0*
**B**	*0*	*1*	*0*	*0*	*0*	*1*	*0*	*0*	*0*	*0*
**C**	*0*	*0*	*1*	*1*	*0*	*1*	*0*	*0*	*0*	*0*
**D**	*0*	*0*	*0*	*1*	*0*	*1*	*0*	*0*	*0*	*0*
**E**	*1*	*1*	*1*	*1*	*1*	*1*	*0*	*1*	*1*	*1*
**F**	*1*	*1*	*0*	*0*	*0*	*1*	*0*	*0*	*0*	*0*
**G**	*1*	*1*	*1*	*1*	*1*	*1*	*1*	*1*	*1*	*1*
**H**	*1*	*1*	*1*	*1*	*0*	*1*	*0*	*1*	*0*	*0*
**I**	*1*	*0*	*0*	*0*	*0*	*1*	*0*	*1*	*1*	*1*
**J**	*1*	*0*	*0*	*0*	*0*	*1*	*0*	*1*	*1*	*1*

(a) When the SSIM (*i, j*) entry was “*X”* both (*i, j*) and (*j, i*) entry in the IRM became 1

(b) When the SSIM (*i, j*) entry was “*O”* both (*i, j*) and (*j, i*) entry in the IRM became 0

(c) When the SSIM (*i, j*) entry was “*A”* the (*i, j*) and (*j, i*) entry in the IRM became 0 and 1 respectively

(d) When the SSIM (*i, j*) entry was “*V”* the (*i, j*) and (*j, i*) entry in the IRM became 1 and 0 respectively

**Table 5:** Final reachability matrix for components of HC

Components	A	B	C	D	E	F	G	H	I	J	Driving
**A**	*1*	*1*	*0*	*0*	*0*	*1*	*0*	*0*	*0*	*0*	3
**B**	1^*^	*1*	*0*	*0*	*0*	*1*	*0*	*0*	*0*	*0*	3
**C**	1^*^	1^*^	*1*	*1*	*0*	*1*	*0*	*0*	*0*	*0*	5
**D**	1^*^	1^*^	*0*	*1*	*0*	*1*	*0*	*0*	*0*	*0*	4
**E**	*1*	*1*	*1*	*1*	*1*	*1*	*0*	*1*	*1*	*1*	9
**F**	*1*	*1*	*0*	*0*	*0*	*1*	*0*	*0*	*0*	*0*	3
**G**	*1*	*1*	*1*	*1*	*1*	*1*	*1*	*1*	*1*	*1*	10
**H**	*1*	*1*	*1*	*1*	*0*	*1*	*0*	*1*	*0*	*0*	6
**I**	*1*	1^*^	1^*^	1^*^	*0*	*1*	*0*	*1*	*1*	*1*	8
**J**	*1*	1^*^	1^*^	1^*^	*0*	*1*	*0*	*1*	*1*	*1*	8
**Dependency**	10	10	6	7	2	10	1	5	4	4	–

1^*^: Entries included to incorporate transitivity

**Table 6:** Level partitioning of components of HC

Components	Reachability set	Antecedent set	Intersection Set	Level
**A**	1,2,6	1,2,3,4,5,6,7,8,9,10	1,2,6	I
**B**	1,2,6	1,2,3,4,5,6,7,8,9,10	1,2,6	I
**C**	1,2,3,4,6	3,5,7,8,9,10	3	III
**D**	1,2,4,5	3,4,5,7,8,9,10	4,5	II
**E**	1,2,3,4,5,6,8,9,10	5,7	5	VI
**F**	1,2,6	1,2,3,4,5,6,7,8,9,10	1,2,6	I
**G**	1,2,3,4,5,6,7,8,9,10	7	7	VII
**H**	1,2,3,4,6,8	5,7,8,9,10	8	IV
**I**	1,2,3,4,6,8,9,10	5,7,9,10	9,10	V
**J**	1,2,3,4,6,8,9,10	5,7,9,10	9,10	V

The MICMAC matrix which was derived from the FRM (Fig. 2) indicated that half of the components including organizational rules and structures, dominant culture of educational environment, teaching and assessment approaches, as well as clinical and educational physical setting were independent or driving factors while the rest were dependent factors.

## Discussion

We conducted this study to identify the main component of HCME and to investigate the interrelationships among them, using ISM. This method helps us to gain better understanding of the concept and its components. The quantitative phase of the study was carried out after a comprehensive review and a panel discussion that could increase validity of the results. After carrying out a scoping review and performing a qualitative content analysis, the key components of HCME were classified into four categories including structural factors, educational factors, cultural factors, and social factors. These findings are largely consistent with the results of another scope study aimed at identifying plural definitions of HCME. The study reported four conceptual boundaries for definition of hidden curriculum including institutional–organizational, contextual–cultural, interpersonal–social, and motivational–psychological aspects [28].

The ISM model (Fig. 1) revealed that basic organizational aspects including rules, structures and culture were the most influential components of HCME. These aspects could determine teaching and assessment methods as well as the structure of clinical and educational environments. Moreover, the model indicated that role modeling behaviors and interpersonal relationships (social factors) are under influence of above-mentioned factors (structural and educational factors). These findings imply that, when HC is investigated as a learning resource, the role of organizational aspects and educational determinants is of highest importance. It can also be concluded that any adjustment in the role modeling functions and also modification of interpersonal relationships with the aim of improving learning outcomes requires changes in the basic organizational and educational variables.

Findings from the ISM model and MICMAC matrix revealed that the “organizational rules and structures” component has the maximum driving power and is the strongest independent factor among the main components of HCME. These facts suggest that other components of HCME are affected by the rules and structures of educational organization. A scoping review on components of HCME indicated that institutional–organizational factors are among the most important components and should be considered appropriately [28]. It is suggested that the structures and rules of educational organization should be modified to replace negative views among students with positive perceptions in order to increase their self-confidence and learning outcomes [15]. Due to the inevitable impact of organizational rules and structures on education outcomes, some efforts have been made by ME experts to modify this important component of HC [35, 36]. However, it is suggested that success of this efforts depends on improving teacher-student relationships [24]. As an example, it has been argued that one of the negative aspects of organizational rules and culture is the assumption that teaching is merely a transfer of concepts and knowledge to the students in order to store in their mind for subsequent retrieval and application [37]. This approach ignores the interpersonal relationships that could enhance learning. Alternate paradigm conceives learning as a constructed approach in which proper teacher-student interaction will help students to construct meaning from ideas and connecting them to the past knowledge, skills, and experiences and making decision about them [38].

This study revealed that the second component with the maximum driving power was organizational culture. It has been reported that the learning of medical students is largely influenced by the dominant culture of educational organization [27]. Unwritten rules, rituals, and customs are the most important aspects of an organization’s culture [22]. The cultural context of educational environments has inevitable positive or negative influences on the learning outcomes [34]. Moreover, it has great impact in professionalism development [39], clinical competency [40], and ethical practice [41]. It is essential to identify the undesirable aspects of organizational culture and modify them to deal with negative educational outcomes [34].

Educational factors including teaching methods and evaluation approaches were identified as other independent or driving components of HCME. Interactive teaching methods have the nature of inductive learning and can enhance future working competencies among medical students [26]. Assessment of students based on problem-solving and decision-making skills far beyond just passing the exams is associated with more active learning and, as a result, more efficient clinical practice [26, 33]. Moreover, it seems that teaching and evaluation methods, especially in clinical settings, may have a role modeling function with high impacts on learning and practice. One important aspect of educational factors is that these components of HC are associated with development of some key values of the medical professionalism such as excellence and leadership [39].

Findings of this study revealed that the last independent or driving component of HCME was educational and clinical physical setting. Clinical setting and university campus have an implicit effect on the socialization of medical students [13]. Some studies have reported that ME environments could be associated with academic achievements and learning outcomes [30, 31]. Since educational environment is influenced by organizational rules, structures, and cultures, educators and educational managers should increase their knowledge and awareness in this regard [25].

Findings of the MICMAC matrix and the ISM model indicated that all social factors as well as the students’ cultural context were dependent factors. The ISM analysis revealed that interpersonal relationships in educational environments and students’ role modeling behaviors were influenced by the basic components of HC such as organizational rules and structures as well as predominant culture of these educational institutions. Some studies have reported that role modeling, through a communication-based learning process, have both positive and negative effects on learning and future practice of medical students [22, 23, 29]. Positive role models have an important effect on the development of professional identity among medical students [22], while bad role models can demotivate students and lead to undesirable learning and practice outcomes [23]. In this regard, it is essential to develop the knowledge of teachers and educators to enhance the desired results [22].

Interpersonal relationships especially between teachers and students play a key role in medical education and effectively determine learning outcomes [38]. Teacher-student relationships is considered as an important source of experience for future practice of medical students [24]. Although the ISM findings showed that interpersonal interaction factors are among the dependent components of HC, it is noteworthy that clinical communications are associated with some important values of professionalism such as respect, responsibility, accountability, altruism, honor, and integrity [39]. Although teacher-student relationships is often seen as a one-way interaction, a number of studies in ME suggested that these interactions take place in a reciprocal process [42, 43]. In addition to the definitive impact of this mutual relationships on students’ learning outcomes, teachers and educators may obtain new insights for future educational practice. This type of communication with a reciprocal influence on both sides could be considered as the relationships between the formal and the hidden curriculum [24]. Eventually, it could be concluded that teachers and educators have a responsibility to modify their relationships with students and patients in accordance with the formal curriculum goals.

## Limitations

At the second phase of this study was applied the ISM method which is based on experts’ opinions. The main limitation of analyzing experts’ opinions is that these types of studies can be strongly influenced by the cultural context. Moreover, the subjective judgments of participants could reduce validity of the results. However, in order to improve validity of the findings, participants of this study attended a discussion panel and informed about the purpose of the study and the method used. Besides, we tried to achieve a consensus on the concepts. Secondly, at the first step of ISM, integrated SSIM developed using the “mode” of experts’ opinions. One of the main limitations in this regard is that the difference between the number of opinions in various pair-wise comparisons and also the initial opinions of participants are not taken in to account. Thirdly, although we tried to carry out a comprehensive review of the literature, it is expected that we may have missed a number of related studies. Another limitation of this study was limited access to ME experts familiar with the concept and the model.

## Conclusion

The scoping review on the key components of HCME led to the development of four main categories including structural factors, educational factors, cultural factors, and social factors. The ISM method showed that the most influential and driving components of HCME were structures and rules of ME organizations, dominant culture of educational environments, teaching and assessment approaches, as well as clinical and educational physical setting. These findings must be considered by ME planners, medical university officials, as well as teachers and educators at all stages of educational management including planning process, implementation of the programs, and development of formal curricula. According to the importance of contextual factors, the main components of HC must be analyzed and interpreted based on the specific conditions of each educational institution.

## Data Availability

The datasets analyzed during the current study are available from the corresponding author on request.

## Abbreviations

HC: Hidden Curriculum
ME: Medical Education
HCME: Hidden Curriculum in Medical Education
ISM: Interpretive Structural Modeling
SID: Scientific Information database
SSIM: Structural Self-Interaction Matrix
RM: Reachability Matrix
IRM: Initial Reachability Matrix
FRM: Final Reachability Matrix
MICMAC: (Matrice d’ Impact Croises- Multiplication Appliquee a un Classement) or (Cross Impact Matrix-Multiplication Applied to Classification)
